# Emotion dysregulation, dissociation and body dissatisfaction mediate the relationship between trauma exposure and ED symptoms

**DOI:** 10.1192/j.eurpsy.2022.1480

**Published:** 2022-09-01

**Authors:** L. Lev-Ari, A. Zohar, R. Bachner Melman

**Affiliations:** 1 Ruppin Academic Center, The Lior Tsfaty Center For Suicide And Mental Pain Studies, Emek Hefer, Israel; 2 Ruppin Academic Center, Clinical Psychology, Emek Hefer, Israel

**Keywords:** Eating disorder symptoms, Emotion dysregulation, Dissociation, Trauma

## Abstract

**Introduction:**

The current study tests the relationship between eating disorder (ED) symptoms and trauma exposure. The mechanisms via which trauma is related to ED symptoms have not been sufficiently examined. This study examines the complex role of dissociation and emotional dysregulation in the context of trauma, BMI, ED symptoms and body dissatisfaction (BD).

**Objectives:**

We hypothesized that dissociation and emotional dysregulation would mediate the relationship between trauma exposure and ED symptoms / BD. We further hypothesized that BMI would play a moderating role in this association.

**Methods:**

A community sample of 229 (16.2% male) participants, with a mean age of 29.08±10.68 reported online on traumatic events (Life Events Checklist), dissociation (Dissociative Experiences Scale – II), emotional dysregulation (Difficulties in Emotional Regulation Scale), ED symptoms (Eating Disorders Examination – Questionnaire) and BD (Figure Rating Scale).

**Results:**

Participants reported experiencing a mean of 2.87±2.27 traumatic events, with a relatively high percentage (˜86%) reporting at least one. The most commonly reported traumatic events were transportation accidents and physical assault. Although frequency of traumatic events did not directly predict ED symptoms, BMI, dissociation, emotional dysregulation and BD did. An SEM model showed that traumatic events predicted ED symptoms indirectly through dissociation, emotional dysregulation and BMI. Dissociation and emotional dysregulation predicted ED symptoms directly. BMI also moderated the association between traumatic events and both ED symptoms and BD.

**Conclusions:**

Therapists treating patients with high BMI or obesity should be aware of these relationships and investigate the possibility that trauma and/or PTSD may underlie the presenting disordered eating or eating disorder.

**Disclosure:**

No significant relationships.

